# The influence of calibration on bias in quality control and patient results for TSH on Vitros XT 7600 analyzer

**DOI:** 10.11613/BM.2024.030703

**Published:** 2024-08-05

**Authors:** Jill Boreyko, Josko Ivica

**Affiliations:** 1 Hamilton Regional Laboratory Medicine Program (HRLMP), Hamilton, Ontario, Canada; 2 Department of Pathology and Molecular Medicine, McMaster University, Hamilton, Ontario, Canada

**Keywords:** TSH, bias, quality management

## Abstract

**Introduction:**

Thyroid-stimulating hormone (TSH) is a glycoprotein secreted by the anterior pituitary gland and is regulated by negative feedback from the serum free thyroid hormones. In this study we aimed to quantitate the relative bias caused by calibration drifting as seen in our TSH Levey-Jennings quality control (QC) charts and assess the magnitude of bias on patients’ samples.

**Materials and methods:**

In the period from October 2021 to August 2022 we looked at the QC results of ten 28-days’ calibration time intervals and calculated relative bias compared to the mean. For each time interval the mean from three QC points before and after calibration was calculated. The average from 10 pre- and post-calibration means was calculated and the relative bias, pre- and post-calibration, was then calculated. We used 5 patient samples with low, normal and high TSH concentrations and calculated relative bias pre- and post-calibration. The allowed relative bias for TSH is ± 6.7%.

**Results:**

At both QC levels, with the respective means of 5.14 mIU/L (coefficient of variation, CV% = 3.1%) and 27.80 mIU/L (CV% = 3.2%) had their respective relative bias - 8.2% and - 7.9%. The patient samples with low (0.586 mIU/L), normal (2.89 mIU/L and 5.19 mIU/L) and high (20.5 mIU/L and 39.8 mIU/L) TSH had - 4.1%, - 4.0%, - 3.5%, - 5.1% and - 4.1%, respectively.

**Conclusion:**

Even though the relative bias exceeded allowable criteria for the QC samples, this was not manifested on the patients’ samples.

## Graphical abstract

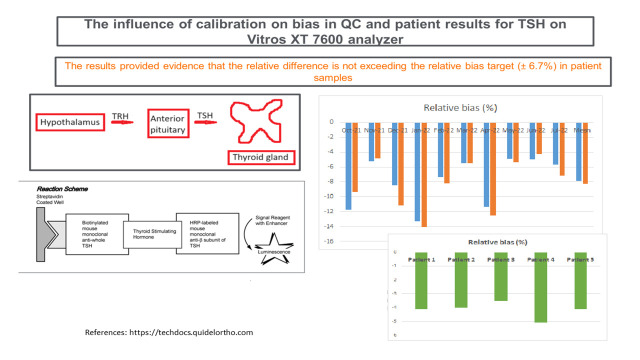

## Highlights

• We aimed to quantitate the relative bias caused by calibration drifting as seen in our TSH Levey-Jennings quality control charts

• We assessed the magnitude of bias on patients’ samples, and compare such bias with the bias target for TSH

• The results provided evidence that the relative difference is not exceeding the goal for the bias in patient samples.

## Introduction

Thyroid-stimulating hormone (TSH) is a hormone secreted by the anterior pituitary gland and its role is to promote secretion of thyroid hormones by the thyroid gland. The thyroid hormones include thyroxine (T4) and triiodothyronine (T3) (*1*-*4*). Minor changes in serum free T4 are reflected by increased TSH secretion by pituitary gland and in developing hypothyroidism and hyperthyroidism the blood concentrations of TSH change remarkably even before free T4 concentration is affected (*2*, *5*). For this reason, TSH is used as a first-line screening test for assessment of thyroid gland dysfunction (*2*, *3*). This is especially made possible with the development of highly sensitive third-generation immunoassays, which are capable of detecting TSH concentrations < 0.01 mIU/L (*5*, *6*).

In clinical laboratories, assays are regularly calibrated to ensure continuous stability, accuracy and reliability of patient results. The calibration frequency is often established by the assay manufacturer or determined in the laboratory based on their practical experience. In addition to those scheduled ones, calibrations are also performed at any one time when it is necessary, *e.g.* when instrument changes occur, when assay does not perform as expected, *etc.* The assay performance and suitability for patients testing is verified by performing necessary quality procedures. Quality control (QC) procedures are integral part of clinical laboratory operations, and are performed at an established frequency, dictated by the laboratory quality management system.

In this study we aimed to quantitate the relative bias caused by calibration drifting as seen in our TSH Levey-Jennings QC charts and assess the magnitude of bias on patients’ samples, and compare such bias with the bias target for TSH.

## Materials and methods

This study was performed in the core laboratory of the Hamilton General Hospital, Hamilton Regional Laboratory Medicine Program (HRLMP), Hamilton, Ontario, Canada. We selected five patient serum samples, one with low, two with high, and two with normal TSH concentrations. All of the samples were de-identified and anonymous. HRLMP reference interval for TSH for adults is 0.47-4.68 mIU/L. Serum samples (Beckton Dickinson, Franklin Lakes, USA) are drawn in 4 mL red top tube without clot activator or any additives by nursing staff using standard operating procedures. The samples are delivered to the core laboratory by the pneumatic tube system. They were let clot for 40 minutes and then centrifuged at 2000xg for 10 minutes. Once the samples were analyzed on our clinical chemistry instrument Vitros XT 7600 (QuidelOrtho, San Diego, USA) they were aliquoted and stored at - 20 °C until their re-analysis. The reagent used for Vitros XT 7600 is an immunometric assay using streptavidin coated well attached to a biotinylated mouse monoclonal anti-whole TSH. The TSH in the sample reacts with anti-whole TSH and it is visualized by adding the horseradish-labeled mouse monoclonal anti-β subunit of TSH. The patient aliquots were thawed and run *prior* calibration (September 13, 2022). Another aliquot was run the same day after TSH has been calibrated. The relative difference between patient TSH results before and after calibration was calculated. The patient results were run on the same calibrator and reagent lot (No. 6780) and from the same reagent bottle.

In our laboratory, TSH QC procedures are performed twice daily and the TSH assay is scheduled to be calibrated every 28 days in case there is no need for an urgent, unscheduled calibration. During this period there were no calibration performed before pre-scheduled 28 days. Two levels of Immunoassay Plus (Bio-Rad Laboratories, Hercules, USA) QC material are used. We use the fixed mean and the fixed standard deviation (SD) to monitor the performance of QC for our patients as our standard procedure. The fixed mean is calculated from 20 points collected for that specific lot of reagent and QC material. After 20 points have been collected, the fixed mean is set until a new reagent lot or QC material is changed. For SD, 6 months’ data are taken from our QC software (Bio-Rad Laboratories, Hercules, CA) for every analyte measured in our laboratory and using mathematical formula, a long-term SD is established. This is revised every 6 months and, if needed, a new SD is re-established.

This project has been approved by the Hamilton Integrated Research Ethics Board (HiREB) on behalf of Hamilton Health Sciences, St. Joseph’s Healthcare Hamilton and McMaster University.

### Statistical analysis

**Figure 1:**
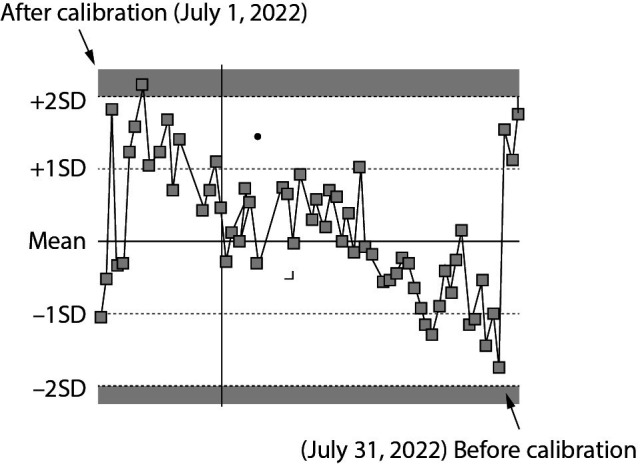
Quality control chart depicting a representative TSH calibration cycle. TSH - thyroid-stimulating hormone.

At both QC levels, with the respective means of 5.14 mIU/L and 27.80 mIU/L the coefficients of variation (CV%) were 3.1% (SD = 0.159 mIU/L) for level 2 and 3.2% for level 3 (SD = 0.090 mIU/L). In order to assess how calibration drift affects QC material, we selected ten 28-days’ time intervals and looked at the patterns in our TSH Levey-Jennings QC charts. The selected time period was from October 2021 to August 2022. During that time two QC lots were used, with the change occurring on May 25, 2022. Both calibrator and reagent lot changed three times (December 12, 2021; March 8, 2022; June 24, 2022) with four lots being used in total. For the purpose of this study, however, we calculated the running means in the individual cycles. Firstly, we looked at the QC results of 10 calibration time intervals and compared to error budget for bias. The same was done with patient samples. For each 28-days’ interval we calculated the mean from three QC points before calibration and the mean from three QC points after calibration. We calculated the average from all 10 pre- and post-calibration means. The relative difference between two means, pre- and post-calibration, was then calculated. We used the running means in this study to assess the relative difference in QC before and after TSH calibration. The running means are the means or averages from actual, measured QC, on the analyzer itself. The mean of three QC points before and after calibration is taken as statistical estimate of the running mean. Figure 1 depicts one such calibration cycle where fixed mean and fixed SD (refer to Materials and methods section for more details on how fixed mean and SD are established) are used. The running means from 3 QC points were calculated in order to assess the relative difference pre- and post-calibration. The bias target typically set in our laboratory is 1/3 of total allowable error (TEa). The QC rules established in our laboratory are 1-3s and 2-2s (s = SD), and if any of these are violated, running the QC or patient samples, is immediately suspended until QC has passed (Figure 1).

## Results

The mean values of the 10 running means for level 2 were 4.7 mIU/L (range: 4.5 mIU/L to 5.0 mIU/L) before calibration and 5.1 mIU/L (range: 4.9 mIU/L to 5.3 mIU/L) after calibration and the mean values for level 3 were 25.6 mIU/L (range: 23.8 mIU/L to 27.3 mIU/L) before calibration and 27.8 mIU/L (range: 26.7 mIU/L to 28.9 mIU/L) after calibration. From these values we calculated the relative difference between pre- and post-calibration running means and the mean relative difference was - 8.2% (- 4.3% to - 14.1%) for level 2 (Table 1) and - 7.9% (- 4.9% to - 13.3%) for level 3 (Table 2).

**Table 1:** TSH results for level 2 quality control samples, before and after calibration

**Bio-Rad Immunoassay Plus Level 2 QC**											**Mean**
Post-calibration	4.986	4.904	5.130	5.187	5.126	5.043	5.342	5.201	5.232	5.277	5.14
Pre-calibration	4.518	4.664	4.560	4.457	4.704	4.767	4.676	4.921	5.008	4.901	4.72
Relative bias (%)	- 9.39	- 4.89	- 11.11	- 14.07	- 8.23	- 5.47	- 12.46	- 5.38	- 4.28	- 7.13	- 8.24
TSH - thyroid-stimulating hormone. QC - quality control.											

**Table 2:** TSH results for level 3 quality control samples, before and after calibration

**Bio-Rad Immunoassay Plus Level 3 QC**											**Mean**
Post-calibration	27.28	26.72	26.82	27.46	27.47	27.33	28.69	28.29	28.74	28.93	27.77
Pre-calibration	24.08	25.33	24.55	23.80	25.44	25.83	25.44	26.89	27.30	27.28	25.59
Relative bias (%)	- 11.73	- 5.20	- 8.46	- 13.32	- 7.39	- 5.49	- 11.33	- 4.95	- 5.01	- 5.70	- 7.86
TSH - thyroid-stimulating hormone. QC - quality control.											

The relative bias between post-calibration and pre-calibration of TSH assay in patient samples was ranging between - 5.1 to - 3.5% (Table 3).

**Table 3:** Patient samples TSH results before and after calibration

**Patient**	**TSH pre-calibration (mIU/L)**	**TSH post-calibration (mIU/L)**	**% relative bias**
1	0.586	0.611	- 4.1
2	2.890	3.010	- 4.0
3	5.190	5.380	- 3.5
4	20.500	21.600	- 5.1
5	39.800	41.500	- 4.1
TSH - thyroid-stimulating hormone.			

## Discussion

In this study we aimed to show whether relative difference seen before and after calibration has an impact on QC and patient samples. In the study performed by Lim *et al.* the authors reported that the variability between calibrations can be larger than the within calibration (CV_between_ / CV_within_ ratio) (*7*). This may not be the case we saw with our assay as we did not see a large shift in the running means but the calibration drift. The drift indicates that the assay is unstable and the more often calibration is warranted. It is very important to understand and appreciate the impact of calibration on analytical systems, where, for example, errors in calibration make little difference in estimating severely decreased estimated glomerular filtration rate (eGFR) (< 30 mL/min/1.73m^2^), but result in progressively larger differences at higher eGFRs (*8*). In another study, the authors investigate the potential impact on health care costs from calibration error resulting in analytical bias in tests to measure serum calcium concentrations (*9*).

There are certain limitations in this study that we need to address. Although calibrator lot hasn’t changed during the course of this project, QC lot has changed twice and the reagent lot has changed three times, with 4 reagent lots being used altogether. These lot changes may have resulted in shifts in means during this experiment and underestimate the effect of relative biases we saw in this experiment. We recognize the limitation of the lack of the stability study in our protocol as well as not using the standard reference material WHO IRP 80/558. Also, we did not include external quality assessment (EQA) samples, in order to assess calibrator uncertainty, which is needed for the expanded measurement uncertainty calculation, especially since our manufacturer did not have information on calibrator uncertainty in their instructions for use. Our goal is that in our future experiments we use all or most of above mentioned limitations not used in this protocol to assess their impact on TSH results.

In conclusion, even though the relative bias exceeded allowable criteria for the QC samples, this was not manifested on the patients’ samples.

## Data Availability

The data generated and analyzed in the presented study are available from the corresponding author on request.
